# Scarlatina without Pyrexia

**Published:** 1910-01-08

**Authors:** 


### SCARLATINA WITHOUT PYREXIA.

There can be little doubt that the type of scarlatina seen nowadays is very different from that which was constantly observed by practitioners upwards of 30 years ago. The disease is upon the whole much modified, much less catching, and much more liable to differ from the type described in the text-books. This no doubt is due in part to the isolation of cases, so that the virulence of the disease has been modified, though it is also probable that even if there had been no isolation of infectious cases this disease, like many other specific fevers, would have spontaneously presented periods of less virulence alternating with periods of greater virulence, just as is seen, for example, in influenza.

One of the abnormal varieties of scarlatina is that in which there is little or no pyrexia, and the following is an example of that condition. A boy, aged 11, was shown by his parents at 8.30 a.m. cn January 21 to the doctor, who had just called to see a sister suffering from acute bronchitis. The lad had complained the night before of a sore throat, but had since slept well and there had been neither nausea nor vomiting, and he was getting ready to go to school when the medical man was asked to see him, merely because he happened to be already in the house. The tonsils, the pillars of the fauces, the edges of the soft palate were all of an intense red colour, without any trace of either exudation or follicular pus. A diagnosis of scarlatinal sore throat suggested itself, and the child's clothes were accordingly removed, when, to the surprise of the parents, the neck, chest, abdomen, and back were found to be covered with a typical scarlatinal punctate erythema. The pulse-rate was, however, only 80, and the temperature but 99.2° F. Notwithstanding the absence of pyrexia, the lad was isolated and put to bed. That same evening at 6 p.m. he was in good spirits and was scoffing at the precautions taken in regard to him, because he said he felt quite well. Nevertheless the eruption had extended considerably since the morning, and was now fully out upon the arms and legs, as well as upon the trunk; the throat was still very red and the tongue was typical of scarlatina; the temperature was 99.2° F. and the pulse-rate 92; the urine was clear and abundant and contained no albumen. The usual treatment of milk diet and antiseptic lavage for the throat was adopted, but no particular medicine was given.

Next day, January 22, the eruption was as intense as before, the urine again abundant and free from albumen, the temperature being 99.2° F. in the morning and 99° F. in the evening, and the pulse-rate 100 in the morning and 106 in the evening. There was no headache, no nausea, and no vomiting, but the sore throat persisted. On January 23 the eruption was beginning to fade, but the throat was still as red as before; the temperature was 99° F. in the morning and evening; the urine remained as before.

On January 24 the eruption had almost completely disappeared, though there were still traces of it in the form of red patches here and there about the body. The pharynx, tonsils, and the pillars of the fauces were still a bright red from the acute injection; the tongue was beginning to lose its fur by desquamation and to assume a deep red colour beneath a glazed surface; the temperature was 98° F. in the morning and 98.6° F. in the evening, the pulse-rate being 100 in the morning and 94 in the evening. The general condition remained excellent.

For the next few days the temperature remained normal, the pulse ranging, however, between 90 and 100; the urine was examined every day, but it never contained albumen; the injection and redness of the tongue and the throat still persisted. From the time that the eruption had faded the boy's body was examined nearly every day, and on February 4, 14 days after the first appearance of the rash, there was the first sign of desquamation upon the elbows; on February 6 the ears and the fingers of both hands were also desquamating, and other parts followed suit. The desquamation was complete by February 17, and the child recovered practically without any complication.

This, then, was a boy who went through his entire attack of scarlatina without having a temperature higher than 99.2° F., and the possibility of this occurring in other cases also is an important point to bear in mind. It seems difficult to deny that the case was one of scarlatina. The disease started with the red, injected sore throat, which preceded the eruption by 24 hours, and the latter had all the points of a scarlatinal eruption; the tongue, after having been furred, peeled, leaving the deep red typical of scarlatina; the redness of the pharynx persisted for a very long time, and finally the desquamation began spontaneously 14 days after the eruption, lasted eight days, and occurred upon the parts which one would expect it to in scarlatina.

Clinically, such cases as these may be even more benign than the above, and may then pass entirely undetected unless some complication ensues, such as otitis media or acute nephritis. Indeed, one wonders whether, in view of this, some of the cases of acute Bright's disease in young children that arise without apparent cause may not be due to very mild and unrecognised attacks of scarlatina.

